# Reduced Insulin-Like Growth Factor 1 Is Associated with Insulin Resistance in Obese Prepubertal Boys

**DOI:** 10.1155/2021/6680316

**Published:** 2021-08-24

**Authors:** Jiangying Kuang, Li Zhang, Yueqin Xu, Jiang Xue, Shuang Liang, Juan Xiao

**Affiliations:** ^1^Department of Cardiology, The Second Hospital, Cheeloo College of Medicine, Shandong University, China; ^2^Child Health Care Center, The First Affiliated Hospital of Shandong First Medical University, China; ^3^Department of Pediatrics, The Second Hospital, Cheeloo College of Medicine, Shandong University, China; ^4^Center of Evidence-Based Medicine, Institute of Medical Sciences, The Second Hospital, Cheeloo College of Medicine, Shandong University, China

## Abstract

As one of the most common features of obesity, insulin resistance is central to the pathogenesis of the metabolic syndrome. Low insulin-like growth factor 1 (IGF-1) levels have been proven to be associated with many traditional cardiovascular risk factors, but it still remains controversial with the relationship between IGF-1 and insulin resistance. Accordingly, the main purpose of this study is to investigate the relationship between IGF-1 and insulin resistance in obese prepubertal boys. We used the whole-body insulin sensitivity index (WBISI) to represent insulin resistance. 70 obese prepubertal boys were included in the study, and the obese subjects were divided into two groups by using 1.285 as a threshold value for WBISI. Clinical examination and laboratory examinations were assessed for all participants. Among obese boys, the group of children with WBISI ≤ 1.285 had lower IGF-1 standard deviation scores (SDS) (*p* = 0.021) than the WBISI > 1.285 group. The results of multiple linear analyses show that lg WBISI was positively correlated with IGF-1 SDS (*p* = 0.031) after adjusting for traditional cardiovascular risk factors. IGF-1 SDS was negatively associated with insulin resistance in obese prepubertal boys, independent of other traditional cardiovascular disease risk markers.

## 1. Background

Childhood obesity has become a major health problem all over the world. It is not only an independent chronic metabolic disease but also an important risk factor for chronic diseases such as hypertension, hyperlipidemia, type 2 diabetes, and metabolic syndrome in children [1]. As the most common feature of childhood obesity, insulin resistance is critical to the pathogenesis of the metabolic syndrome. Several quantitative tools have been used to assess insulin sensitivity and insulin resistance [2], but there is still no consensus of standards in the pediatric population. Although hyperinsulinemic-euglycemic clamp and the frequently sampled intravenous glucose tolerance test were described as gold standard methods for assessing insulin resistance, it is impractical to be widely used in pediatric population due to its expensive and multiple blood drawn. The homeostatic model assessment index of insulin resistance (HOMA-IR), a simpler, less invasive method, has been widely used in children and adolescents; however, Shaibi et al. [3] showed that fasting indices are not recommended when studying the effect of interventions on insulin sensitivity in overweight youth. The whole-body insulin sensitivity index (WBISI) derived from an oral glucose tolerance test (OGTT) provides reasonable estimates of insulin sensitivity and insulin resistance and has been validated as good surrogate measures of insulin resistance in obese children and adolescents [4, 5].

Insulin-like growth factor 1 (IGF-1) is primarily produced in the liver upon stimulation by growth hormone (GH), and it plays key roles in regulating proliferation, differentiation, metabolism, and cell survival [6]. There were lots of accumulating evidences that obese children and experimental animals were often accompanied with low IGF-1 levels [7, 8]. Moreover, low IGF-1 levels were independently associated with cardiovascular risk factors such as nonalcoholic fatty liver disease [9], low high-density lipoprotein cholesterol [7], and metabolic syndrome [7] in obese children. Nonetheless, it still remains controversial with the relationship between IGF-1 and insulin resistance. Furthermore, most previous studies used adults as main samples, and also, most of them just used HOMA-IR to represent insulin resistance. To the best of our knowledge, no study has ever used WBISI to represent insulin resistance to assess the relationship between IGF-1 and insulin resistance in children. To this end, this study is aimed at investigating the association between IGF-1 and WBISI in obese prepubertal boys.

## 2. Materials and Methods

### 2.1. Subjects

We recruited 70 obese prepubertal boys (age 7–12 years) who had been referred to the Department of Pediatrics, The Second Hospital, Cheeloo College of Medicine, Shandong University. The inclusion criteria: all subjects were obese (body mass index (BMI) > 95th percentile for the age and sex), but otherwise healthy. All participants were prepubertal boys. None had any syndrome or disease that could influence dietary intake and endocrine disorders. None had type 1 or 2 diabetes mellitus, serious infection, systemic disease, and other chronic wasting illnesses. None had short stature, or the growth velocity is less than 5 cm/year. None was taking medication that would influence body composition, GH secretion or blood pressure, glucose, or lipid metabolism, and no one has any nutritional and supplements intake.

Taking WBISI as the main variable, obese children were classified according to the median WBISI values to find the center value for this population of obese children. Thus, the obese children were divided into two groups: obese children with a WBISI ≤ median value and obese children with a WBISI > median value.

The Ethics Committee of The Second Hospital, Cheeloo College of Medicine, Shandong University approved the study. Written informed consent was obtained from all parents and subjects.

### 2.2. General Information and Anthropometric Measurements

General information includes family economic status and parents' education level. We used parents' monthly household income to represent family economic status. Monthly household income based on the following 3-point level: 1 ≤ ¥5000, 2 = ¥5001 ~ ¥10,000, and 3 ≥ ¥ 10,001 (¥ is the unit of RMB). Parents' education level is based on the following 3-point level: 1 = college education not completed, 2 = completed college education, and 3 = completed graduate education. Body weight was determined to the nearest 0.1 kg on a standard electronic scale, and height was measured with a standard height stadiometer (TJ-220S-18, Beideneng, Shanghai, China), to the nearest 0.1 cm. BMI was determined as weight/height^2^ and expressed as kg/m^2^. Body mass index standard deviation score (BMI-SDS) was calculated based on the age and sex reference values for Chinese children [10]. The pubertal stage is according to Tanner criteria [11]. Blood pressure was measured with an Audio Intelligent Electronic Sphygmomanometer (HEM-7071, OMRON, China) after a 30 min rest, in a supine position. Two measurements were made, and record the average of two measurements of systolic blood pressure (SBP) and diastolic blood pressure (DBP).

### 2.3. Laboratory Measurements

Fasting blood samples were collected from subjects after a 12 h overnight fast for measurement of endocrine indexes, glucose, lipid levels, and other metabolic factors. OGTT was performed. Plasma glucose and insulin values were assessed at time 0, 30, 60, 90, and 120 minutes after the consumption of an oral glucose solution (1.75 g/kg, maximum of 75 g). Free triiodothyronine (FT3), free thyroxine (FT4), thyroid-stimulating hormone (TSH), adrenal corticotropic hormone (ACTH), cortisol (COR), and IGF-1 were measured using chemiluminescence assay (Siemens Healthcare Diagnostics, USA). The intra- and interassay CVs were 5–9%. IGF-1 levels were transformed into IGF-1 SDS based on the age-gender-related normative references [12], and the intra- and interassay CVs were 4.5% and 7.7%, respectively. Total cholesterol (TC), high-density lipoprotein cholesterol (HDL-C), low-density lipoprotein-cholesterol (LDL-C), triglycerides (TG), fasting plasma glucose (FPG), C-reactive protein (CRP), and uric acid were detected by using an Auto Biochemical Analyzer (AU5400, Beckman Coulter, Tokyo, Japan). Fasting insulin was measured with chemiluminescent immunometric assays (CobasE170, Roche Diagnostics, Mannheim, Germany). Plasma glycosylated hemoglobin (HbA1c) was measured using high-performance liquid chromatography (Tosoh Corporation, Tokyo, Japan). The intra-assay and interassay coefficients of variation were <8.0%. Insulin resistance was estimated using WBISI. WBISI = 10,000/square root of [fasting glucose × fasting insulin{*μ*IU/mL}] × [mean glucose{mg/dL} × mean insulin during OGTT{*μ*IU/mL}] [13]. We also calculated other indicators for evaluating insulin sensitivity/resistance: HOMA-IR, homeostasis model assessment of *β*-cell function (HOMA-beta), and insulinogenic index (IGI). HOMA-IR is defined as fasting insulin (*μ*IU/mL) × fasting glucose(mmol/L)/22.5. HOMA-beta is defined as fasting insulin (*μ*IU/mL) × 20)/[fasting glucose(mmol/L)–3.5]. IGI = [30 minute insulin–fasting insulin(*μ*IU/mL)/[30 minute glucose–fasting glucose(mg/dL)].

Impaired fasting glucose (IFG)was defined as FPG ≥ 5.6 to 6.9 mmol/L. Impaired glucose tolerance (IGT) was defined as a 2 h blood glucose between 7.8 and 11.1 mmol/L [14]. The metabolic syndrome (MS) was defined as the presence of any three or more of the following five constituent risks according to the criteria of the International Diabetes Federation [15]: (1) abdominal obesity, (2) HDL‐C < 1.03 mmol/L, (3) TG ≥ 1.7 mmol/L, (4) hypertension, (5) IFG.

### 2.4. Statistical Analysis

We used the Statistical Package for Social Sciences, version 20.0 (SPSS Inc. Chicago, USA) to analyze our data. The data were expressed as the mean (±SD) of normally distributed or median (interquartile range) of skewed data. Data that were not normally distributed were transformed logarithmically for analysis. Differences in continuous variables between two groups were assessed by Student's *t*-test for normally distributed data; variables which cannot be transformed to normal distribution were analyzed by the Mann-Whitney *U* test. The relationships among lg WBISI and other variables were evaluated based on the methods of Pearson's correlation analysis or Spearman correlation analysis. Variables that achieved statistical significance in the univariate analysis (including IGF-1 SDS, lg insulin, and FBG) were subsequently included in a multiple linear regression model. A stepwise multiple linear regression analysis was performed to test the associations of lg WBISI (the dependent variable) with independent variables to determine whether the association between IGF-1 SDS and WBISI was independent of other markers. A *p* value of <0.05 was considered statistically significant.

## 3. Results

The median WBISI value for the population studied was 1.285. The data of the children with WBISI ≤ 1.285 (35 children) were compared with the data of the children with WBISI > 1.285 (35 children).

The characteristics of the anthropometric and clinical features are shown in Table 1. No significant differences were found in family economic status, parents' education level, and age. BMI SDS was similar in the two groups. Blood pressure values and the incidence of FPG, IGT, and MS were the same in both groups.

In obese children, pituitary-thyroid axis, pituitary-adrenal axis, and IGF-1 have been performed. Endocrine factor findings of the obese children were stratified according to WBISI, which are shown also in Table 1. Notably, the group of children with WBISI ≤ 1.285 had lower IGF-1 SDS (*p* = 0.021). No significant differences were seen in the levels of FT3, FT4, TSH, ACTH, and COR in the two groups.

Table 1 also shows that the levels of cardiovascular metabolic risk factors of the obese children were stratified according to WBISI. No significant differences were seen in the levels of CRP, TC, HDL-C, LDL-C, TG, and uric acid between the two groups. In addition, there were no significant differences in FBG and HbA1c, whereas higher insulin levels, glucose 120 min, insulin 120 min, HOMA-IR, HOMA-beta, and IGI (all *p* < 0.001) were observed in the subjects with WBISI ≤ 1.285 than in children with WBISI > 1.285.

We performed a bivariate correlation analysis to explicit the relationship between lg WBISI and other variables in study population (Table 2). lg WBISI was positively associated with IGF-1 SDS (*r* = 0.358, *p* = 0.002) but was negatively associated with FBG (*r* = −0.394, *p* = 0.001) and lg insulin (*r* = −0.723, *p* < 0.001). However, lg WBISI were not significantly associated with other variables.

Univariate analysis showed that IGF-1 SDS, FBG, and lg insulin could be selected for the final equation. Furthermore, results from the multiple linear regression analysis showed that lg WBISI was positively correlated with IGF-1 SDS (*p* = 0.031) and was negatively correlated with lg insulin and FBG (*p* < 0.001, *p* = 0.006). The results of multiple regression analysis showed that IGF-1 SDS, lg insulin, and FBG were the independent determinant of lg WBISI in obese prepubertal boys after adjusting for other variables (shown in Table 3).

## 4. Discussion

Our study found that reduced IGF-1 were present in obese prepubertal boys with lower values of WBISI, which is a surrogate marker of insulin resistance. Furthermore, we provided evidences that WBISI was significantly correlated with IGF-1 SDS after controlling for other metabolic risk factors.

In the study, we confirmed a significant association between low levels of IGF-1 and insulin resistance in obese prepubertal boys. Moreover, the results of multiple regression analysis showed that independent correlation between insulin resistance is represented by WBISI and IGF-1 SDS after adjusting for other traditional cardiovascular disease risk markers. Based on the results, we can infer that the correlation between IGF-1 and traditional cardiovascular disease may be mediated by insulin resistance; thus, it represents a possible direction for a forthcoming research and therapeutic approach.

As we mentioned in the preface, several studies have evaluated the correlation between IGF-1 and insulin resistance in adults [16–21]. The results of most previous studies were consistent with our results [16–18, 20]. By contrast, a U-shaped curve [19] or no significant correlation [21] was noted between IGF-1 levels and insulin resistance in other studies. The possible reasons for these differences were that these studies reflect differences in study population and assess methods in insulin resistance. In our study, we choose WBISI to represent insulin resistance, which is considered to be a good surrogate measure of insulin resistance in obese children; it should have a certain scientific and clinical significance.

There is still not very clear about the precise underlying mechanisms of the correlation between IGF-1 and insulin resistance. The possible reasons of this correlation may be related to the following factors. Insulin and IGF-1 share structural homology, which interact with the same membrane receptors with different affinities to mediate a wide range of metabolic and growth-promoting functions. Studies have shown that insulin was likely to decrease IGF-1 through differential modulation of IGF-binding proteins [22]. This may be one mechanism for the link of IGF-1 and insulin resistance. Another possible explanation for the association between IGF-1 and insulin resistance is driven by chronic inflammatory. Low levels of IGF-1 in the C3H.6T mice [23] and in the liver-specific IGF-1 knockout mouse [22] were associated with enhanced inflammatory phenotype. On the contrary, elevated IGF-1 levels could attenuate the anti-inflammatory effects and the lowered oxidative stress [24–26]. Meanwhile, inflammatory factors and chronic inflammatory responses play an important role in the occurrence and development of insulin resistance [27].

In addition, macrophages may also be involved in the complex process of interaction between IGF-1 and insulin. Insulin has been considered to be proinflammatory in macrophages [28] and has been reported to promote foam cell formation [29]. By contrast, recent research showed that IGF-1 might have a fundamental role in macrophage activation [30, 31] and had the anti-inflammatory [31] and antifoam cell formation [32] effects. Further studies are necessary to clarify specific mechanisms and effects of IGF-1 and insulin on macrophages.

This study also has several potential limitations. Firstly, we have not applied hyperinsulinemic-euglycemic clamp which is considered to be the gold standard for assessing insulin resistance. However, the clamp technique is invasive, expensive, and not suitable for child clinical practice. Secondly, it is a cross-sectional study, so the causal relationship between IGF-1 and insulin resistance cannot be clarified. Thirdly, another limitation of our research is that we lacked the normal control group, but parents cannot accept repeated blood tests on their healthy children. Failure to evaluate participants' food intake and physical activity is another limitation of our research. Lastly, our sample was limited to Chinese obese prepubertal boys, but gender and pubertal developmental stage restriction can also reduce confounding factors. In spite of these limitations, to the best of our knowledge, this study is one of the first attempts to report the association between IGF-1 and WBISI in obese children; thus, it should have some implications for clinical practice. Our data suggested that the assessment of IGF-1, performing OGTT experiments, and calculating WBISI might provide useful clinical information on the severity and complications of obesity in children.

In conclusion, we have found a strong correlated relationship between low IGF-1 and insulin resistance in obese prepubertal boys independently of other risk factors. The role of IGF-1 needs further examination whether it is a potential therapeutic target in obese patients of increased cardiometabolic risk or only an indicator of metabolic disorders. And further studies are needed to determine the characteristics of the pathophysiology of the association between IGF-1 and insulin resistance in obese children and obesity-related complications.

## Figures and Tables

**Table 1:** Clinical characteristics in the two groups according to WBISI levels.

Variable	WBISI ≤ 1.285 (*n* = 35)	WBISI > 1.285 (*n* = 35)	*p* value
Family economic status			0.350^#^
≤¥5000/monthly	8/35	11/35	
¥5001 ~ ¥10,000/monthly	18/35	12/35	
≥¥10,001/monthly	9/35	12/35	
Maternal education			0.668^#^
<college	6/35	6/35	
≥college	17/35	17/35	
≥graduate	12/35	12/35	
Paternal education			1^#^
<college	8/35	11/35	
≥college	12/35	12/35	
≥graduate	15/35	12/35	
Age (yr)	11.18 ± 1.51	10.71 ± 2.09	0.291
FPG	11/35	5/35	0.077^#^
IGT	7/35	3/35	0.153^#^
MS	18/35	16/35	0.406^#^
BMI SDS	3.35 ± 0.91	3.12 ± 0.16	0.298
SBP (mmHg)	125.57 ± 15.49	125.40 ± 13.57	0.961
DBP (mmHg)	81.54 ± 11.74	78.14 ± 12.03	0.236
IGF-1 SDS	−1.08 ± 1.78	−0.22 ± 1.25	0.021^∗^
FT3 (pmmol/L)	6.37 ± 0.96	6.07 ± 0.72	0.151
FT4 (pmmol/L)	16.20 (14.67-17.34)	14.69 (12.24-16.72)	0.068
TSH (*μ*U/mL)	2.94 (1.87-3.42)	3.79 (2.02-5.40)	0.076^#^
ACTH (mmol/L)	31.85 (17.47-49.24)	22.80 (16.46-39.29)	0.144^#^
COR (pg/mL)	330.00 (264.30-534.00)	360.00 (241.00-453.00)	0.539
TC (mmol/L)	4.31 (3.77-4.68)	4.16 (3.56-4.67)	0.156
HDL-C (mmol/L)	1.11 ± 0.21	1.12 ± 0.24	0.850
LDL-C (mmol/L)	2.64 (2.26-2.99)	2.51 (16.46-39.29)	0.162
TG (mmol/L)	1.39 (0.89-2.22)	1.30 (0.85-1.75)	0.557
Uric acid (*μ*mol/L)	422.49 ± 102.33	404.78 ± 131.30	0.531
Insulin (*μ*IU/mL)	52.17 (39.20-60.70)	17.40 (14.10-26.36)	<0.001^∗^
FBG (mmol/L)	5.24 ± 0.67	4.99 ± 0.47	0.078
Glucose 120 min (mmol/L)	6.97 (6.48-7.37)	6.11 (5.35-6.92)	<0.001^∗^^#^
Insulin 120 min (*μ*IU/mL)	273.80 (193.90-475.90)	56.96 (20.91-136.10)	<0.001^∗^
HOMA-IR	12.12 (8.12-14.06)	3.87 (3.12-6.05)	<0.001^∗^
HOMA-beta	673.00 (431.02-865.53)	252.79 (174.67-355.05)	<0.001^∗^
HbA1c (%)	5.90 (5.70-6.30)	5.80 (5.50-6.00)	0.473
IGI	4.26 (3.01-6.37)	2.12 (1.38-3.28)	<0.001^∗^
CRP	3.12 (1.07-4.61)	2.92 (1.14-5.68)	0.933

^#^Chi-square test or Mann-Whitney *U* test; ^∗^*p* < 0.05.

**Table 2:** Bivariate correlation analysis between lg WBISI and other variables in study population.

Variable	*r*	*p*
Age (yr)	-0.017	0.891
BMI SDS	-0.052	0.670
SBP (mmHg)	-0.098	0.419
DBP (mmHg)	-0.159	0.189
IGF-1 SDS	0.358	0.002^∗^
TSH (*μ*U/mL)	0.158	0.190
ACTH (mmol/L)	-0.167	0.166
COR (pg/mL)	-0.107	0.378
lg TC (mmol/L)	-0.076	0.530
HDL-C (mmol/L)	0.041	0.735
LDL-C (mmol/L)	-0.039	0.747
lg TG (mmol/L)	0.012	0.922
Uric acid (*μ*mol/L)	-0.147	0.226
lg insulin (*μ*IU/mL)	-0.723	<0.001^∗^
FBG (mmol/L)	-0.394	0.001^∗^

^∗^*p* < 0.05.

**Table 3:** Multivariate linear regression analysis for the determinant of lg WBISI in obese prepubertal boys.

Constant	*B*	*t*	*p* value
IGF-1 SDS	0.037	2.199	0.031^∗^
lg insulin	-0.752	-7.313	<0.001^∗^
FBG	-0.123	-2.643	0.006^∗^

^∗^*p* < 0.05.

## Data Availability

The datasets used and/or analyzed during the current study are available from the corresponding author on reasonable request.

## Abbreviations

HOMA-IR: Homeostasis model assessment-insulin resistance
OGTT: Oral glucose tolerance test
WBISI: Whole-body insulin sensitivity index
IGF-1: Insulin-like growth factor 1
GH: Growth hormone
BMI: Body mass index
BMI SDS: Body mass index standard deviation scores
SBP: Systolic blood pressure
DBP: Diastolic blood pressure
FT3: Free triiodothyronine
FT4: Free thyroxine
TSH: Thyroid-stimulating hormone
ACTH: Adrenal corticotropic hormone
COR: Cortisol
TC: Total cholesterol
HDL-C: High-density lipoprotein cholesterol
LDL-C: Low-density lipoprotein cholesterol
TG: Triglycerides
FBG: Fasting blood glucose
CRP: C-reactive protein
HbA1C: Glycosylated hemoglobin
HOMA-beta: Homeostasis model assessment of *β*-cell function
IGI: Insulinogenic index
IFG: Impaired fasting glucose
IGT: Impaired glucose tolerance
MS: Metabolic syndrome.

## References

[1] Skinner A. C., Perrin E. M., Moss L. A., Skelton J. A. (2015). Cardiometabolic risks and severity of obesity in children and young adults. *The New England Journal of Medicine*.

[2] Gutch M., Kumar S., Razi S., Gupta K., Gupta A. (2015). Assessment of insulin sensitivity/resistance. *Indian Journal of Endocrinology and Metabolism*.

[3] Shaibi G. Q., Davis J. N., Weigensberg M. J., Goran M. I. (2011). Improving insulin resistance in obese youth: choose your measures wisely. *International Journal of Pediatric Obesity*.

[4] Brar P. C., Koren D., Gallagher P. R., Pendurthi B., Katz L. E. (2013). Comparison of oral and intravenous glucose tolerance test derived sensitivity and secretory indices in obese adolescents. *Clinical Pediatrics (Phila)*.

[5] Yeckel C. W., Weiss R., Dziura J. (2004). Validation of insulin sensitivity indices from oral glucose tolerance test parameters in obese children and adolescents. *The Journal of Clinical Endocrinology and Metabolism*.

[6] Troncoso R., Ibarra C., Vicencio J. M., Jaimovich E., Lavandero S. (2014). New insights into IGF-1 signaling in the heart. *Trends in Endocrinology and Metabolism*.

[7] Liang S., Hu Y., Liu C., Qi J., Li G. (2016). Low insulin-like growth factor 1 is associated with low high-density lipoprotein cholesterol and metabolic syndrome in Chinese nondiabetic obese children and adolescents: a cross-sectional study. *Lipids in Health and Disease*.

[8] Ren J., Anversa P. (2015). The insulin-like growth factor I system: physiological and pathophysiological implication in cardiovascular diseases associated with metabolic syndrome. *Biochemical Pharmacology*.

[9] Liang S., Cheng X., Hu Y., Song R., Li G. (2017). Insulin-like growth factor 1 and metabolic parameters are associated with nonalcoholic fatty liver disease in obese children and adolescents. *Acta Paediatrica*.

[10] Li H., Ji C. Y., Zong X. N., Zhang Y. Q. (2009). Height and weight standardized growth charts for Chinese children and adolescents aged 0 to 18 years. *Zhonghua er ke za zhi = Chinese Journal of Pediatrics*.

[11] Tanner J. M., Whitehouse R. H. (1976). Clinical longitudinal standards for height, weight, height velocity, weight velocity, and stages of puberty. *Archives of Disease in Childhood*.

[12] Isojima T., Shimatsu A., Yokoya S. (2012). Standardized centile curves and reference intervals of serum insulin-like growth factor-I (IGF-I) levels in a normal Japanese population using the LMS method. *Endocrine Journal*.

[13] Matsuda M., Defronzo R. A. (1999). Insulin sensitivity indices obtained from oral glucose tolerance testing: comparison with the euglycemic insulin clamp. *Diabetes Care*.

[14] American Diabetes Association (2021). 2. Classification and diagnosis of Diabetes:Standards of Medical Care in Diabetes-2021. *Diabetes Care*.

[15] Zimmet P., Alberti K. G. M., Kaufman F. (2007). The metabolic syndrome in children and adolescents ? an IDF consensus report. *Pediatric Diabetes*.

[16] Mannino G. C., Greco A., de Lorenzo C. (2013). A fasting insulin–raising allele at IGF1 locus is associated with circulating levels of IGF-1 and insulin sensitivity. *PLoS One*.

[17] Teppala S., Shankar A. (2010). Association between serum IGF-1 and diabetes among U.S. adults. *Diabetes Care*.

[18] Lam C. S., Chen M. H., Lacey S. M. (2010). Circulating insulin-like growth factor-1 and its binding protein-3: metabolic and genetic correlates in the community. *Arteriosclerosis, Thrombosis, and Vascular Biology*.

[19] Friedrich N., Thuesen B., Jorgensen T. (2012). The association between IGF-I and insulin resistance: a general population study in Danish adults. *Diabetes Care*.

[20] Song X., Teng J., Wang A., Li X., Wang J., Liu Y. (2016). Positive correlation between serum IGF-1 and HDL-C in type 2 diabetes mellitus. *Diabetes Research and Clinical Practice*.

[21] Mallea-Gil M. S., Ballarino M. C., Spiraquis A. (2012). IGF-1 levels in different stages of liver steatosis and its association with metabolic syndrome. *Acta gastroenterologica Latinoamericana*.

[22] Akanji A. O., Smith R. J. (2012). The insulin-like growth factor system, metabolic syndrome, and cardiovascular disease risk. *Metabolic Syndrome and Related Disorders*.

[23] Shai S. Y., Sukhanov S., Higashi Y., Vaughn C., Rosen C. J., Delafontaine P. (2011). Low circulating insulin-like growth factor I increases atherosclerosis in ApoE-deficient mice. *American Journal of Physiology. Heart and Circulatory Physiology*.

[24] Sivasubramaniyam T., Schroer S. A., Li A. (2017). Hepatic JAK2 protects against atherosclerosis through circulating IGF-1. *JCI Insight*.

[25] Sukhanov S., Higashi Y., Shai S. Y. (2011). Differential requirement for nitric oxide in IGF-1-induced anti-apoptotic, anti-oxidant and anti-atherosclerotic effects. *FEBS Letters*.

[26] Jeschke M. G., Barrow R. E., Herndon D. N. (2000). Insulinlike growth factor I plus insulinlike growth factor binding protein 3 attenuates the proinflammatory acute phase response in severely burned children. *Annals of Surgery*.

[27] Shoelson S. E., Herrero L., Naaz A. (2007). Obesity, inflammation, and insulin resistance. *Gastroenterology*.

[28] Manowsky J., Camargo R. G., Kipp A. P., Henkel J., Püschel G. P. (2016). Insulin-induced cytokine production in macrophages causes insulin resistance in hepatocytes. *American Journal of Physiology. Endocrinology and Metabolism*.

[29] Park Y. M., R Kashyap S., A Major J., Silverstein R. L. (2012). Insulin promotes macrophage foam cell formation: potential implications in diabetes-related atherosclerosis. *Laboratory Investigation*.

[30] Higashi Y., Sukhanov S., Shai S.-Y. (2016). Insulin-like growth factor-1 receptor deficiency in macrophages accelerates atherosclerosis and induces an unstable plaque phenotype in apolipoprotein E–deficient mice. *Circulation*.

[31] Higashi Y., Gautam S., Delafontaine P., Sukhanov S. (2019). IGF-1 and cardiovascular disease. *Growth Hormone & IGF Research*.

[32] Sukhanov S., Snarski P., Vaughn C. (2015). Insulin-like growth factor I reduces lipid oxidation and foam cell formation via downregulation of 12/15-lipoxygenase. *Atherosclerosis*.

[33] Kuang J., Zhang L., Xu Y., Xue J., Liang S. (2020). Association between insulin-like growth factor 1 and insulin resistance in obese prepubertal boys: a cross-sectional study.

